# Supplementary material for: High school science fair: Positive and negative outcomes
Source: PLoS One. 2020 Feb 13;15(2):e0229237. doi: 10.1371/journal.pone.0229237 (PMC7018130; doi:10.1371/journal.pone.0229237)
Supplement: S1 Survey — (PDF) [file pone.0229237.s004.pdf]

High School Science Fair Survey

Scientists at UT Southwestern Medical Center and Southern Methodist University are interested in learning about student experiences with high school science fair, and we ask for your assistance. We would like to know about your experience and opinions about the help you received in preparing your science fair project and the obstacles you faced and how you overcame them. This survey, which consists of 21 questions, is anonymous; we only are interested in overall trends. You may leave blank any question you prefer not to answer. It is important that you give honest replies because the results may be used to influence science fair practices in the future. Although your participation is voluntary, the more students who participate the more valuable the conclusions will be for possible further action. Thank you for your help.

Enter your 6-digit survey access number

____

---

1. What grade are you in?

◯ 9th
◯ 10th
◯ 11th
◯ 12th

---

2. Gender?

◯ Female
◯ Male

---

3. During high school have you carried out science fair more than once?

◯ Yes
◯ No

If you carried out science fair more than once, then on subsequent questions use your most recent experience to answer.

---

4. Was your science fair project Team or Individual?

◯ Team
◯ Individual

---

5. Was the science fair project required by your school?

◯ Yes
◯ No
◯ No, but I did a science fair project to satisfy a school project requirement.

---

6. Do you think science fair projects should be optional or required? (This need not be for competition.)

◯ Optional
◯ Required

---

7. Reason why?

____

---

8. Do you think science fair projects for competition should be optional or required?

◯ Optional
◯ Required

---

9. Reason why?

____

10. From whom do you think it would be reasonable to receive help on a science fair project?<br>Check all that apply	☐ 1. Parents<br>☐ 2. Siblings<br>☐ 3. Other family members (uncles, cousins, etc.)<br>☐ 4. Teachers<br>☐ 5. Other students<br>☐ 6. Scientists<br>☐ 7. A paid mentor<br>☐ 8. Articles on the Internet<br>☐ 9. Articles in books or magazines<br>☐ Other
Specify:	__________________________________
11. Who actually helped you?<br>Check all that apply	☐ 1. Parents<br>☐ 2. Siblings<br>☐ 3. Other family members (uncles, cousins, etc.)<br>☐ 4. Teachers<br>☐ 5. Other students<br>☐ 6. Scientists<br>☐ 7. A paid mentor<br>☐ 8. Articles on the Internet<br>☐ 9. Articles in books or magazines<br>☐ Other
Specify:	__________________________________
12. What kind of help on a science fair project do you think would be reasonable to expect from others?<br>Check all that apply	☐ 1. Being given the main idea<br>☐ 2. Development of the idea<br>☐ 3. Gathering background research information, or finding a research site or participants<br>☐ 4. Performing the experiments<br>☐ 5. Writing the report<br>☐ 6. Fine tuning the report after it is written<br>☐ 7. Designing the poster board and presentation<br>☐ 8. Producing charts or graphs<br>☐ 9. Coaching for the interview with judges<br>☐ 10. Copying the project from someone else<br>☐ Other
Specify?	__________________________________
13. What kind of help did you actually receive?<br>Check all that apply	☐ 1. Being given the main idea<br>☐ 2. Development of the idea<br>☐ 3. Gathering background research information, or finding a research site or participants<br>☐ 4. Performing the experiments<br>☐ 5. Writing the report<br>☐ 6. Fine tuning the report after it is written<br>☐ 7. Designing the poster board and presentation<br>☐ 8. Producing charts or graphs<br>☐ 9. Coaching for the interview with judges<br>☐ 10. Copying the project from someone else<br>☐ Other
Specify:	__________________________________

14. Did you get the kind of help you wanted from teachers?	◯ Yes<br>◯ No
15. Was there some kind of help that you would have liked but did not receive?<br>Specify:	______________________
16. Did you get the amount of help you wanted from teachers?	◯ Yes<br>◯ No
17. Were the results of your project as expected?	◯ Yes<br>◯ No
18. What obstacles did you face?<br>Check all that apply	☐ 1. Coming up with the main idea<br>☐ 2. Getting motivated to do the project<br>☐ 3. Becoming disappointed with the project<br>☐ 4. Limited resources<br>☐ 5. Limited knowledge<br>☐ 6. Limited skills<br>☐ 7. Limited cooperation<br>☐ 8. Getting organized<br>☐ 9. Time pressure<br>☐ 10. Not enough money<br>☐ 11. Results not as expected<br>☐ Other
Specify?	______________________
19. How did you overcome the obstacles you encountered?<br>Check all that apply	☐ 1. Used someone else's main idea<br>☐ 2. Picked a familiar/interesting topic<br>☐ 3. Did more background research<br>☐ 4. Stopped working on the project for a while<br>☐ 5. Made a timeline to follow<br>☐ 6. Perseverance and self-discipline<br>☐ 7. Had someone else to keep me on track<br>☐ 8. Had someone else do the math<br>☐ 9. Changed the research plan<br>☐ 10. Collected more data<br>☐ 11. Had someone else collect the data<br>☐ 12. Used someone else's data<br>☐ 13. Made up the data<br>☐ 14. Changed the hypothesis to fit the data<br>☐ 15. Changed the data to fit the hypothesis<br>☐ Other
Specify?	______________________
20. Are you interested in a career in the sciences or engineering?	◯ Yes<br>◯ No<br>◯ Not sure
21. Did your science fair experience increase your interest in the sciences or engineering?	◯ Yes<br>◯ No
