# Supplementary figures and images for: High school science fair: Positive and negative outcomes
Source: PLoS One. 2020 Feb 13;15(2):e0229237. doi: 10.1371/journal.pone.0229237 (PMC7018130; doi:10.1371/journal.pone.0229237)

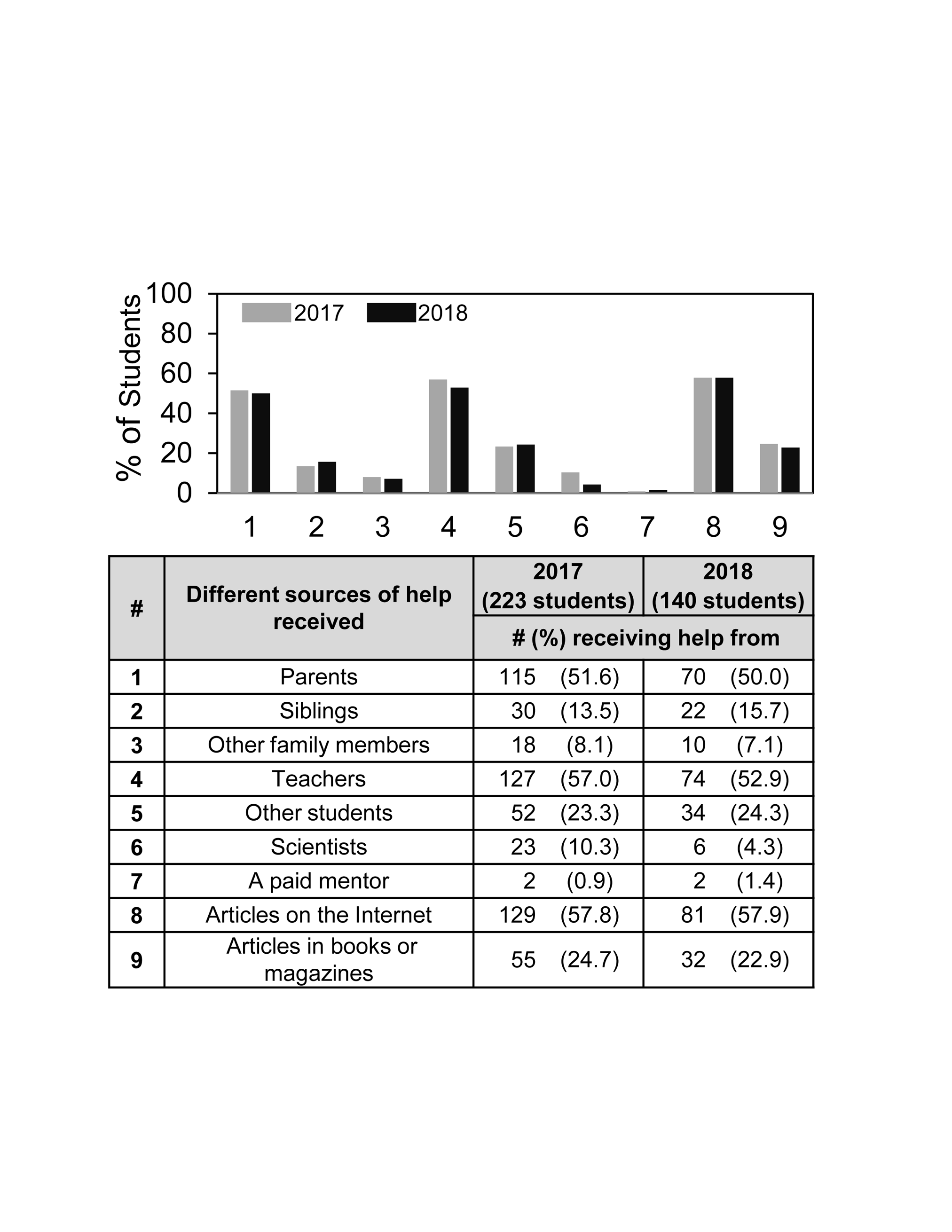

Supplement: S1 Fig — (TIF) [file pone.0229237.s005.tif]

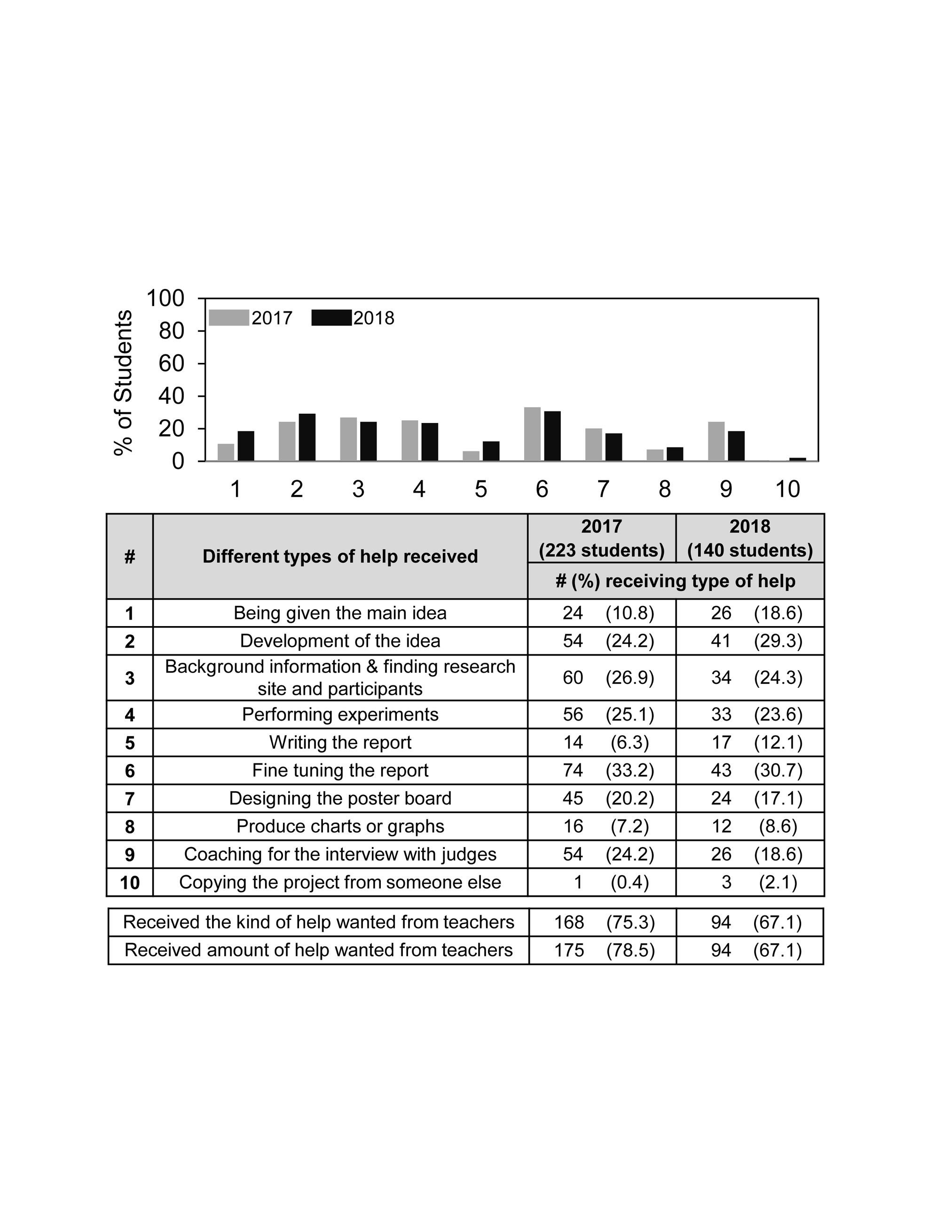

Supplement: S2 Fig — (TIF) [file pone.0229237.s006.tif]

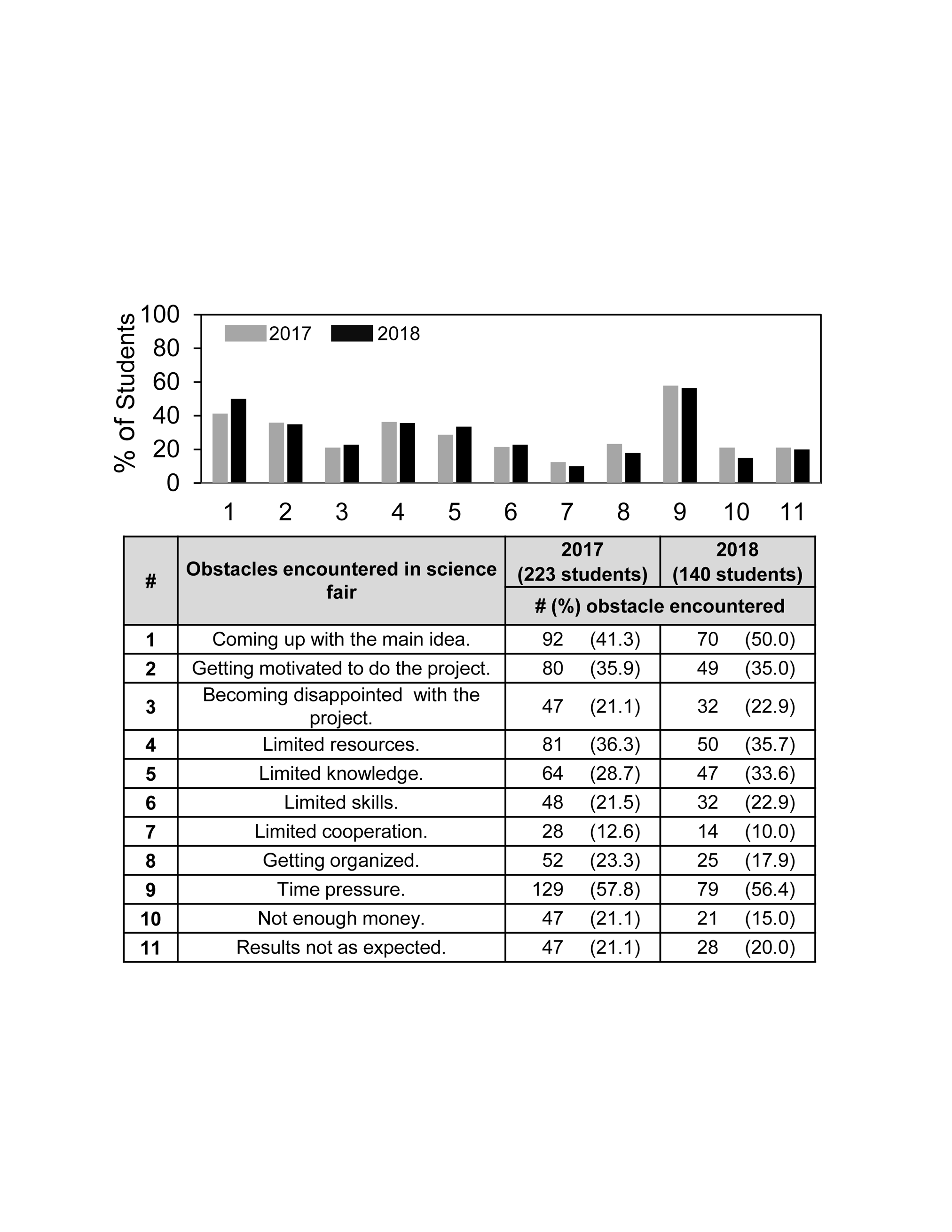

Supplement: S3 Fig — (TIF) [file pone.0229237.s007.tif]

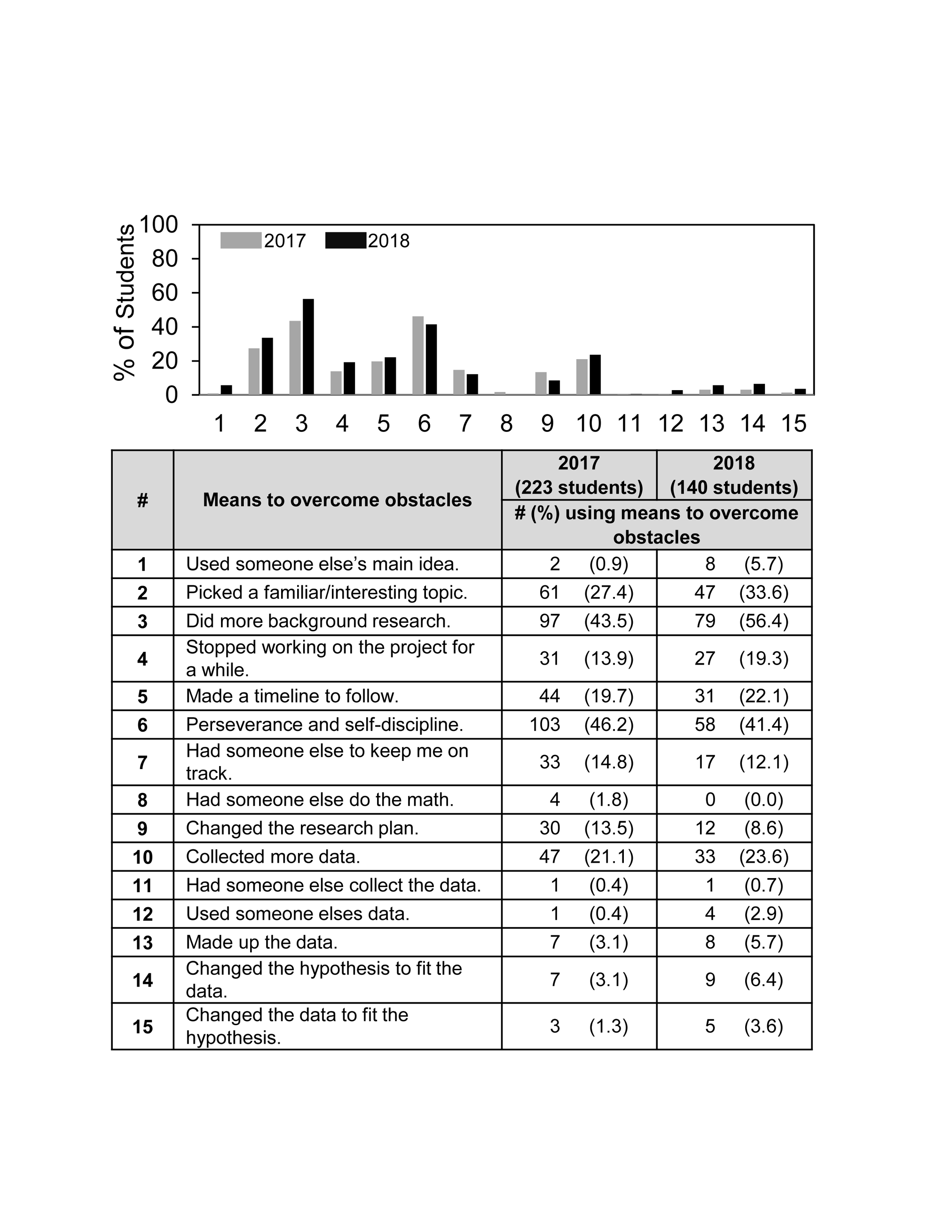

Supplement: S4 Fig — (TIF) [file pone.0229237.s008.tif]

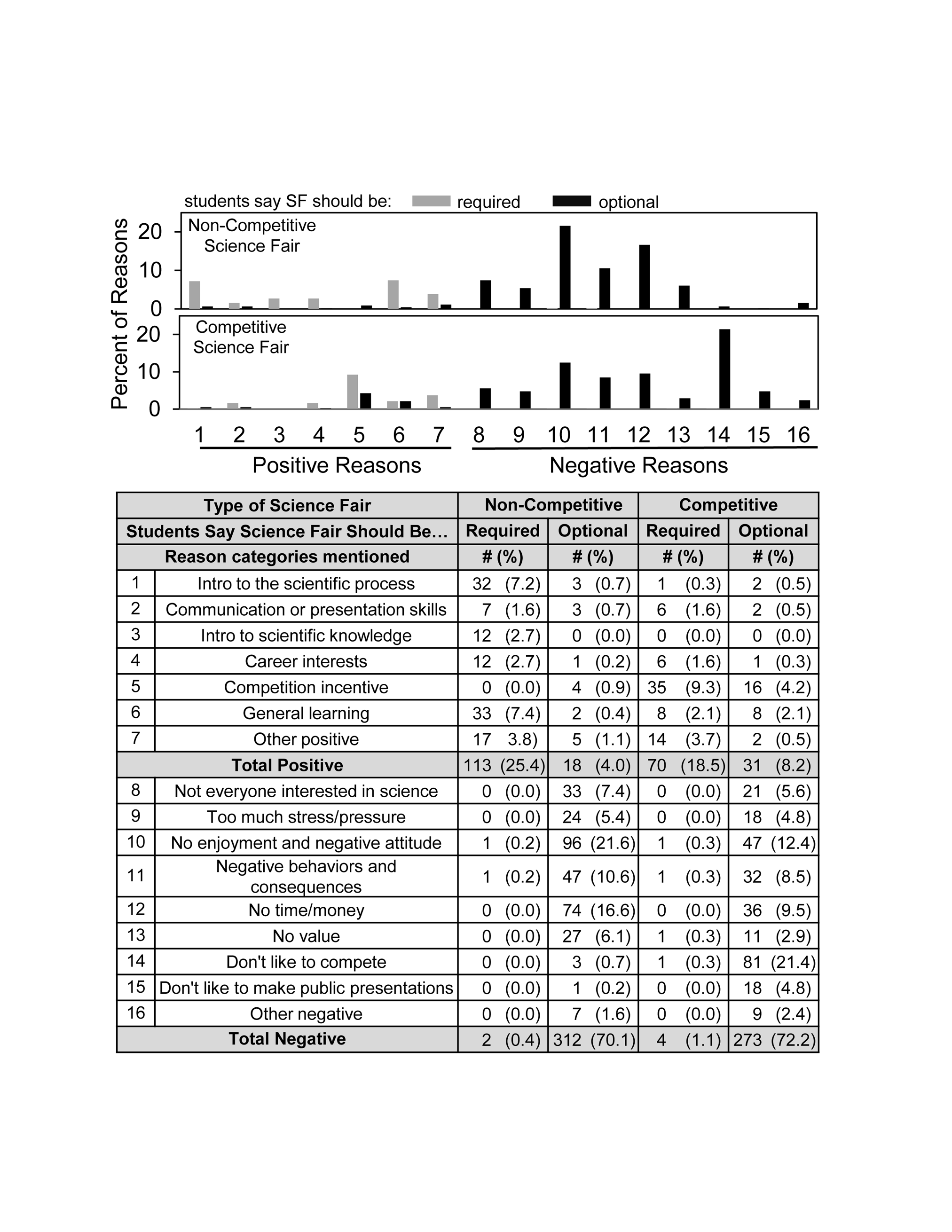

Supplement: S5 Fig — (TIF) [file pone.0229237.s009.tif]
